# Leçons De Physiologie Expérimentale, Appliquée La Médecine, Faites Au Collége De France, &c.

**Published:** 1857-09

**Authors:** 


ART. VII.—*Leçons de Physiologie Expérimentale, Appliquée la Médecine, faites au Collége de France.* Par M. CLAUDE BERNARD, Membre de l'Institut de France. Prof. de Médecine au Collége de France. Prof. de Physiologie à la Faculté des Sciences, &c. &c. Tome Deuxième. Cours du Semestre d'été, 1855. Avec 78 figures intercalées dans le texte.

ON page 545 of the last number of this journal, we alluded to M. Bernard's test for pancreatic tissue and juice, when in certain stages of decomposition. At the time, we spoke, in a note, of the color tests by chlorine and acid, as unreliable, and hinted at the possibility of there being omissions in the text. Since then, we have read a monograph on the pancreas by the same author, but of a later date, in which this portion of the subject is more fully treated. The determination of the anatomical nature of a tissue by means of its chemical reactions, is to some degree novel, and we have had the curiosity to repeat our author's researches in this direction, to some extent confirming his views, and yet satisfying ourselves that at present, to say the least, these tests have but little practical utility, however interesting they may be in their mere chemical relations. In the monograph referred to,

M. Bernard describes the fresh pancreatic tissue or juice, as being distinguishable from salivary, and all other tissues and juices, by the power which they possess of emulsifying and acidulating oils and fluid fats, as we have previously stated. This test is a simple one : a morsel of suspected tissue is rubbed up with a little oil, on a plate, and litmus water put in contact with it. After half an hour or more, fatty acids separating, redden the blue litmus, if the tissue used is pancreatic.

Again, when the pancreas decomposes, says M. Bernard, it is capable of being turned a wine-red by chlorine water. After further decay has occurred this agent no longer acts, and the reddening will then take place only by contact with sulphuric and nitric acids, mixed in proportions of three to one. The chlorine test is available, it seems, after two days; temperature not mentioned. The acid test can only be used later; meanwhile, the albuminoid matters of the gland lose by degrees the power of coagulating with the agents which usually so affect them.

M. Bernard seems to have had no trouble in obtaining the color test, by merely mixing the two fluids; but, as we have had more difficulty at all times, we will state briefly the most certain mode of employing the test. The pancreas may be cut up, crushed with an equal bulk or more of water, and after twenty-four hours filtered, the clear filtrate being left then to decompose. Sometimes we have allowed the gland to decompose alone, and then formed from it a putrid infusion for testing. Either mode answers, and in either case the fluid obtained is more or less coagulable, as it is less or more decomposed. After many days, the odor becomes almost insupportable, vibrias fill the liquid, and it becomes green and opaque. To use the test, the acid or chlorine is placed in a test tube, and the fluid poured upon it in equal amount, and so gently, that the two fluids may meet, but not freely mingle. If the fluids are shaken up together, the test with us, at least, has always failed. When decomposition has set in fully, the test is not affected by previously boiling the pancreas infusion, and using then the clear filtrate.

M. Bernard's chlorine test arose, we fancy, out of an experiment of Tiedemann and Gmelin, who found that an aqueous solution of the dried Alch. extract of a dog's pancreas, became red with chlorine water. We have found, however, such difficulty with the chlorine test, as to have obtained the red tint but twice, and then feebly, in upwards of twenty attempts. It must, therefore, present some difficulties which do not belong to the acid test. The successful cases were with the infusions of a cat's pancreas, five days after death. The chlorine always precipitated some albumen, and in most cases produced a yellow tinge, but no red color.

With the acid test we have had a better success, and have found that in some cases, with certain tissues, nitric acid alone, or its mixture with sulphuric, or even pure muriatic acid, would answer perfectly well. In no case, however, did the color occur under seven days, even in a summer temperature of from 70° to 90° Fahr. In most cases, it could then be observed for periods of from nineteen to thirty days. In no case did we observe the *wine red* described by M. Bernard. The prevailing tint was usually a beautiful *rose red*, or pink, and sometimes such a variety of colors were seen, as present themselves when nitric acid is added to bile.

We shall detail briefly some of the results which we observed, premising, that in some cases we failed to obtain any color test whatsoever. As the object is to discriminate the pancreas from other parts, we have usually tested the salivary glands, blood, muscles, spleen, liver, &c.

Thus, the blood of a large sturgeon was allowed to stand for six days, at a temperature of from 70° to 90° Fahr. The clot redissolved, as it usually does in this fish; and the blood, which was partially decomposed, was then boiled with an equal bulk of water, and filtered. The clear filtrate was set aside for testing. The muscular tissue, the liver, and the spleen, were similarly treated; infusions gave no result with chlorine water save slight coagulation and yellow tints. With nitric and nitro-sulphuric acids, the liver gave only a deep yellow and greenish hue, while with the blood, spleen, and muscle infusions, each acid yielded an exquisite rose pink, with a yellow layer at the line of contact; muriatic acid gave the same result with the blood alone. Usually, this yellow hue was above the rose tint, the mixture finally becoming yellow throughout. Similar results were obtained, in numerous experiments with the various tissues of the cat, the sheep, ox, calf, &c.

Perhaps the pancreas of the sheep answers best, and it is worthy of remark, that muriatic acid yields with its infusion a royal purple, and not a blue, its ordinary color reaction with albumen. Sulphuric acid alone does not answer perfectly well with the pancreas infusion, usually affording a yellow or brown, but no pure rose tints. Nitric acid alone, we have found to be best adapted to most tissues.

Since we have, in every case, failed to observe any red tints in infusions of decomposed salivary tissues, and have generally obtained them in the pancreas, there is here again a point of distinction between these seemingly allied tissues. The test, however, if it deserves the name, is only obtainable by skilful manipulation. It is sometimes perfectly unsuccessful in the pancreas of one sheep, for example, and sufficiently easy and plain in that of another. Lastly, we have obtained very pale rose tints, in decomposing solutions of ordinary egg albumen, kept for twelve days, at summer heat. Here, as with the pancreas solutions, the albumen becomes less and less coagulable as decomposition advances; and here, unlike the case of the pancreas, pure sulphuric acid answers as well, or better, than nitro-sulphuric or nitric acid alone.

